# Leaf thermotolerance in tropical trees from a seasonally dry climate varies along the slow-fast resource acquisition spectrum

**DOI:** 10.1038/s41598-017-11343-5

**Published:** 2017-09-12

**Authors:** Aniruddh Sastry, Deepak Barua

**Affiliations:** Department of Biology, Indian Institute of Science Education and Research, Pune, India

## Abstract

Knowledge of the upper limits of temperature tolerance is essential to understand how tropical trees will respond to global warming. We quantified leaf thermotolerance in 41 tree species growing in a seasonally dry tropical region of the Indian subcontinent to examine: (1) differences between evergreen and deciduous species; (2) relationships with leaf mass per area (LMA) and leaf size; and, (3) seasonal variation in thermotolerance. Thermotolerance ranged from 45.5 °C to 50.5 °C among species, was higher for evergreen than deciduous species, and was negatively related to a continuous estimate of deciduousness. Species with higher LMA had higher thermotolerance, but we did not detect any relationship between leaf size and thermotolerance. Seasonal changes in thermotolerance varied among species implying that species’ capacity to acclimate may differ. Thermal safety margins, the difference between thermotolerance and maximum habitat temperatures indicate that most species may be highly vulnerable to future warming. Overall our results show that deciduous, and fast growing species with low LMA are likely to be more negatively affected by global warming. This differential vulnerability may lead to directional changes in composition in dry tropical forests, and such changes could alter vegetation-atmosphere feedbacks and further exacerbate global warming.

## Introduction

Understanding tolerance to extreme temperatures is essential to assess the vulnerability of species to global warming. The frequency and severity of extreme temperatures are predicted to increase in future climates^1–3^, and exposure to extreme temperatures is likely to have severe consequences for plants, threatening the survival of sensitive species, resulting in local extinctions, range shifts, and altered vegetation composition. This in turn could alter ecosystem structure, function, services and land-atmosphere interactions^4–6^. Tropical systems may be particularly vulnerable to future warming^7^. Tropical species are already living closer to their absolute thermal maxima, and likely have narrower thermal niche breadths due to long term adaptation to relatively stable temperatures^8, 9^. Recent studies have stressed that understanding the responses of tropical forests to increased temperatures is a major limitation in predicting responses to climate change^10–12^. Currently our knowledge of the upper temperature limits of tropical plants comes from a limited number of studies (Supplementary Fig. S1, Supplementary Table S1), and with the exception of the Australian tropics which is marginally better represented, only a dozen studies in about as many sites have examined thermotolerance in tropical trees.

A recent global study showed that thermotolerance of photosynthetic and respiratory function in over 200 trees from seven biomes increases with habitat temperatures^13^. However, the increase in mean thermotolerance from the poles to the equator was ~8 °C, and was much lower than the 20 °C difference in maximum habitat temperatures at these sites. Thus, thermal safety margins, the difference between upper limits of tolerance and maximum habitat temperatures are lower for species from lower latitudes, as also shown for arid regions plants from Australia^14^. Additionally, variation in thermotolerance in co-occurring species within a site is substantial and can range from 10–20 °C^13, 15–19^. Phylogenetic constraints^20^, and heterogeneity in microhabitat conditions^14^ have been proposed as possible explanations, but we do not fully understand the reasons for the shallow relationship between habitat temperatures and thermotolerance, or the large variation within co-occurring species.

Currently, it is not known whether thermotolerance differs between plant functional types, or if it is related to functional traits. If so, species loss due to global warming may not be random and could result in directional changes in community level functional traits, composition, and resultant ecosystem processes and properties^6, 12, 21^. For example, leaf and stem functional traits are important mediators of feedback with the atmosphere, and such directional changes could modify this feedback and alter rates of future climate change^10^. Thus, knowledge of the relationship between thermotolerance and plant functional types and traits will help in predicting vegetation responses to global warming, and understanding the functional consequences of such responses^22^.

Broad-leaved evergreen and dry-deciduous trees are important plant functional types in tropical dry forests^23, 24^. Evergreen species maintain a significant portion of their canopy through the year, have a conservative resource acquisition strategy, and lower productivity^23, 25^. In contrast, dry deciduous species are drought avoiders that shed most of their leaves during the dry season, but have an exploitative resource acquisition strategy with higher photosynthetic rates and water use when water is abundant. Leaves of evergreen species are exposed to a wider range of environmental extremes, have greater structural investment^23, 26^, and have higher tolerance to drought^24, 27^. However, it is not known whether the need to maintain leaves through the year, and the consequent exposure to wider ranges of temperatures might also result in higher thermotolerance in evergreen than in deciduous trees.

Leaf structural and morphological traits modulate relationships between leaf and air temperature, and can determine the range and maxima of temperatures experienced^28–31^. For example, lower leaf size reduces differences between leaf and air temperatures, and is likely to be adaptive in hot and dry environments^31^. Recent empirical and theoretical work suggests that leaf thermoregulation may be extensive^32, 33^, and that leaf temperature may be fundamentally related to leaf traits and carbon economics^34^. Leaf mass per area (LMA) is a key functional trait associated with the tradeoff between resource acquisition and leaf longevity that defines the “slow-fast” leaf economics spectrum^35, 36^. Species with higher LMA, reflective of higher structural investment in leaf tissue, have longer longevity, but lower leaf nitrogen concentrations, photosynthetic rates and growth^35, 36^. Based on the covariance between LMA and traits important for moderating leaf thermal properties, Curtis *et al*. (2012) predicted that LMA should be positively related to thermotolerance. However, results from the few studies that have examined LMA-thermotolerance relationships are equivocal with some suggesting positive relationships^37, 38^, while others have failed to detect any^13, 39^.

Thermotolerance of plants is influenced by recent environmental conditions and increases within individuals with exposure to higher temperatures, reduced water availability and high light intensities^40, 41^. This can result in seasonal changes in thermotolerance which likely represent acclimatory responses^40, 42^. Such acclimatory changes may be particularly pronounced in seasonal environments like dry tropical forests, and the capacity to acclimate may be important in determining how species respond to future warming^13^. However, seasonal changes in thermotolerance have not been examined in a large number of co-occurring species to ask how this may vary among species.

In this study we quantified leaf temperature tolerance in 41 tropical tree species growing in a seasonally dry region in the Indian subcontinent to examine: (1) differences in thermotolerance between evergreen and deciduous species; (2) the relationship of thermotolerance to key leaf functional traits - leaf mass per area and leaf size; and, (3) seasonal variation in thermotolerance between the hot-dry and the cool-wet seasons. We measured the temperature response of Photosystem II (PSII) function - dark-adapted Chlorophyll *a* fluorescence, and quantified the temperature (T_50_) at which PSII function is reduced to 50% of control levels as an index of leaf temperature tolerance. To better understand the relationship of thermotolerance to leaf habit and leafing behaviour, we used monthly estimates of leaf canopy to quantify the average loss of canopy by these species through the year. This provided a continuous estimate of deciduousness (deciduousness index) that is more informative than the discrete evergreen and deciduous categories. Finally, to understand the consequences of variation in thermotolerance among species we estimated thermal safety margins to assess how close the upper limits of tolerance are to maximum temperatures experienced in this region currently, and are likely to experience with future global warming.

## Materials and Methods

### Study site and species

**Figure 1:**
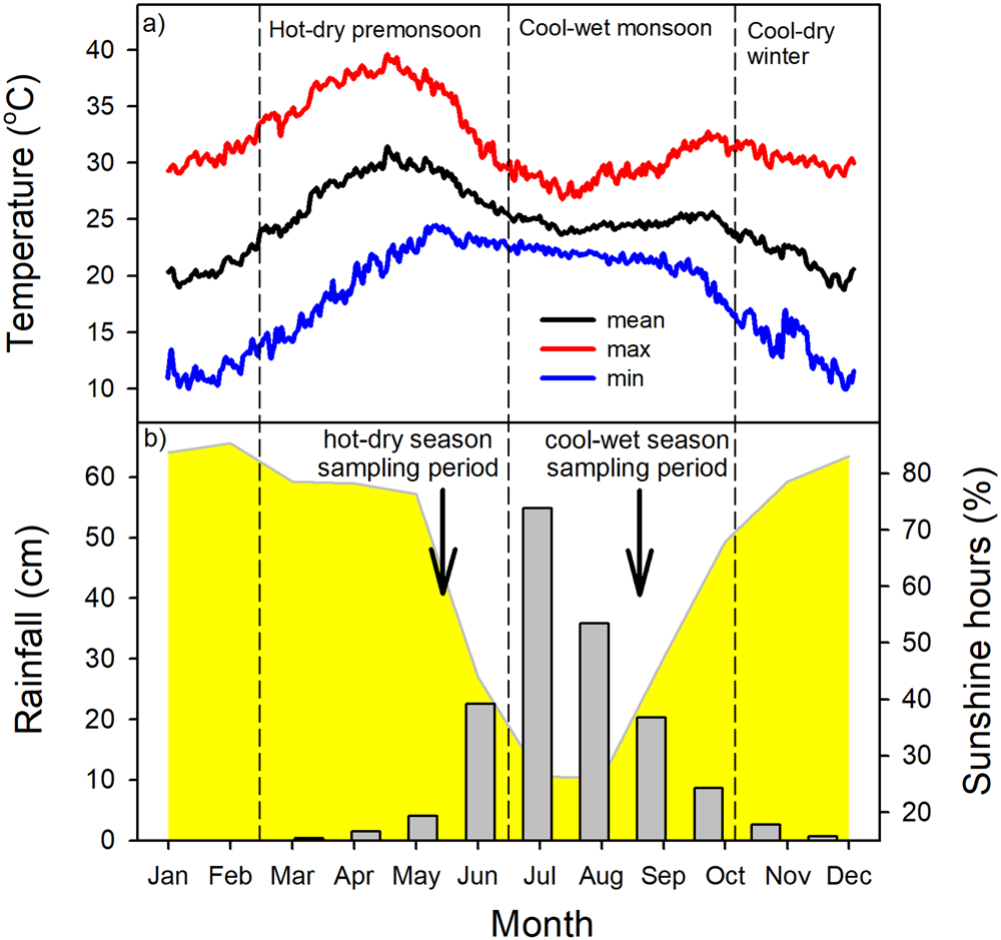
Climate data for the study site (Pune, Maharashtra, India): (**a**) Average daily minimum (blue), maximum (red), and mean (black) air temperatures (2006–2015). Data from GHCN (Global Historical Climatology Network) daily Version 3.22; (**b**) Monthly averaged precipitation (1961–1990) - grey vertical bars; and, sunshine duration (yellow curve). Precipitation and sunshine data are from a high resolution global dataset^57^. The vertical dashed lines demarcate the distinct seasons in the study region - hot-dry pre-monsoon (March–June), cool-wet monsoon (July–October), and cool-dry winter (November–February). Arrows indicate the hot-dry and cool-wet sampling times.

This work was conducted in Pune, Maharashtra, India; in the Baner-Pashan and Pashan (Panchvati) parks (urban parks of ~80 ha each); and the campuses of the National Chemical Laboratory, and the Indian Institute of Science Education and Research which cover ~160 ha (18.541°N, 73.803°E, 560 m ASL). We examined 41 tree species commonly found in this area (Supplementary Table S2). Rainfall in this region is highly seasonal and ~90% of the annual average rainfall of 1516 mm falls between June and October (Fig. 1). Average monthly minimum temperatures in January are around 11 °C, while average monthly maximum temperatures in April are around 37 °C. The absolute high temperature recorded in the last decade was 42.1 °C.

### Phenology monitoring and estimation of deciduousness index

Leaf phenology was monitored for 10 established and reproductively mature individuals of each species from April 2014 to March 2015. For 8 species, 10 individuals were not available and 4–8 individuals were monitored (Supplementary Table S3). Phenology was not monitored for 4 species due to unavailability of sufficient individuals that could be accessed through the year. Censuses were conducted between the 12^th^ and 15^th^ of every month on the same individuals. Phenology observations were initiated 3 months before the final study duration to calibrate and standardize estimates for each species, and conducted by the same observer to avoid observer bias.

Deciduousness was scored by visual estimation of the canopy in a semi-quantitative manner from 0–100% in steps of 10, where 0 represents full canopy, and 100 represents complete leaflessness. The monthly measures of deciduousness were averaged over the year to obtain a deciduousness index (DI) for species. The foliage was further partitioned into flushing, mature, and senescing leaves based on size, colour and texture of leaves. Species for which individuals lost 80% or more of their leaves (excluding senescing leaves) at any time during the year were classified as deciduous, while other species were classified as evergreen.

### Collection of leaf samples and quantification of leaf traits

Leaf samples were collected in 2014, between 28^th^ May and 8^th^ June (dry season), and between 2^nd^ and 13^th^ September (monsoon season). Collection dates in the dry season were selected to ensure that species had flushed leaves and that these were mature at the time of collection (see Supplementary Table S3 and Fig. S2 for peak flushing and senescing details for species). The first fully expanded, and mature leaves from the upper sun-exposed canopy that were free from visible damage from herbivory and pathogens were collected from 4–6 individuals of every species. A telescopic leaf pruner (8 m) was used to access the leaves from the canopy. Leaves were placed in sealed plastic bags with moistened tissue, and were transported to the lab within an hour for quantification of leaf traits and thermotolerance.

Leaf area was measured by scanning fresh leaves with a desktop scanner. Leaf discs were punched with a cork borer (0.8 cm radius), and discs placed in paper bags in a hot-air oven at 70 °C for 3–4 days till a constant dry weight was obtained. Leaf mass per area (LMA) was estimated as the ratio of dry weight to the surface area of leaf discs, from 5 separate leaves per individual, from five replicate individuals of each species (total of 25). LMA for compound leaves were quantified as the average LMA of a leaflet.

### Temperature tolerance assays

We measured the temperature response of dark adapted chlorophyll *a* fluorescence, the maximum potential quantum yield of photosystem II (PSII)^43^. Dark adapted fluorescence is the ratio of variable and maximum fluorescence, *F*_*v*_ */F*_*m*_, where *F*_*v*_ = *(F*_*m*_ − *F*_*o*_), and *F*_*m*_ and *F*_*o*_ are the maximum and basal fluorescence yield, respectively. This physiological measure is an indicator of the integrity of the photosynthetic machinery, is particularly thermolabile, and represents a sensitive indicator of photosynthetic and organismal thermotolerance^41, 44^.

Leaf discs (0.8 cm radius) from 4–6 individuals of every species were used for the assays. The entire leaflet was used for species with compound leaves, where leaflet size was smaller than the leaf punch. Leaf discs were placed between two layers of muslin cloth, covered with aluminium foil and put in a sealed plastic bag with moist tissue at the bottom. This was immersed in a temperature controlled refrigerated water bath (Julabo, Model F25, Seelbach, Germany) preset to the desired temperature (25 °C, 35 °C, 40 °C, 45 °C, 47.5 °C, 50 °C or 52.5 °C) for 30 min. We chose 30 min exposure durations as preliminary experiments, and previous studies^45^ showed that this resulted in irreversible damage to the leaves with negligible recovery after 24 hours. Temperatures of dummy leaf discs (not used for assays) were monitored with a thermocouple attached to the underside of the leaf. Preliminary trials were conducted to determine the temperature of the water bath required to maintain the desired leaf temperatures. Following the 30 min exposure to treatment temperatures, the leaf discs were allowed to dark adapt at room temperature for an additional 30 min before dark adapted chlorophyll *a* fluorescence (*F*_*v*_ */F*_*m*_) was measured with a PAM 2500 fluorometer (Walz, Effeltrich, Germany).

**Figure 2:**
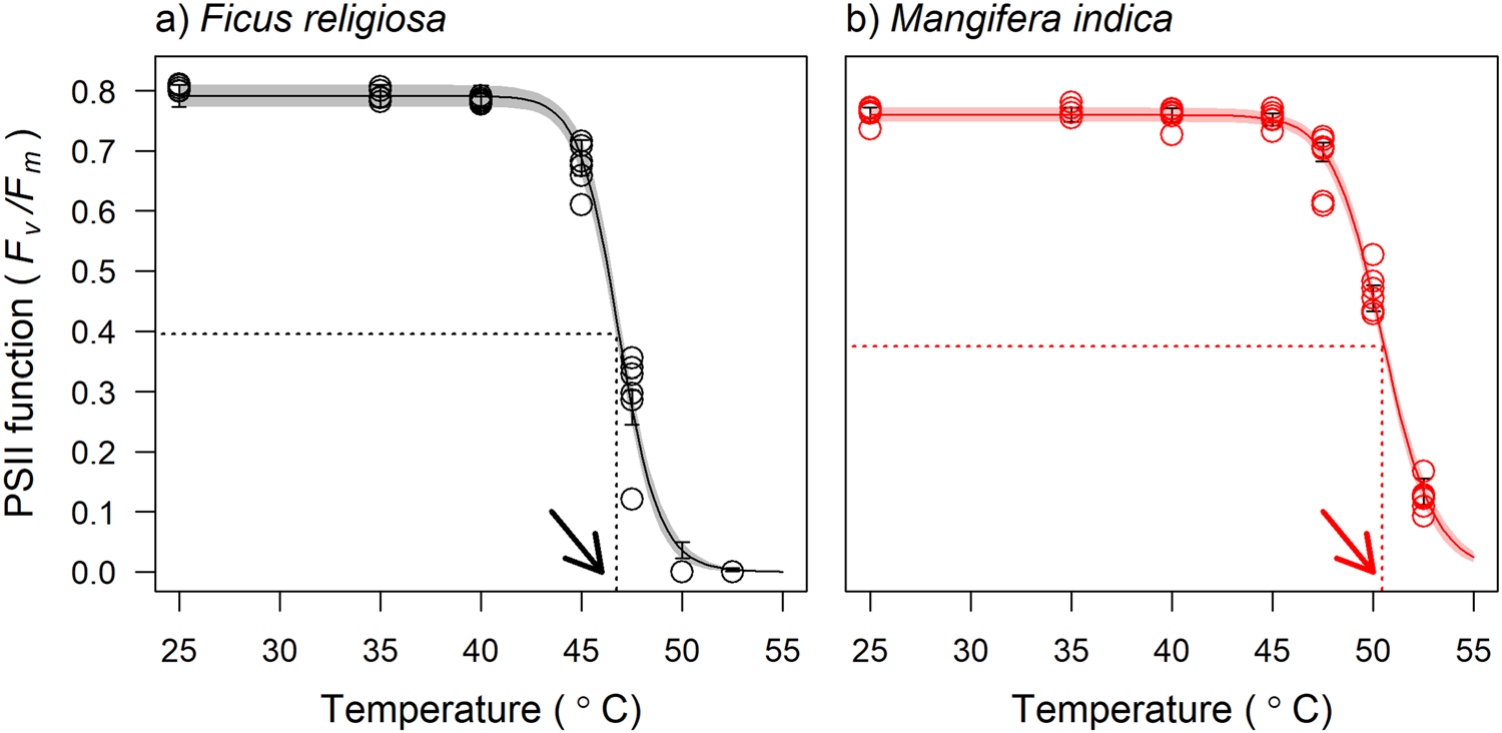
Representative species level temperature response curves for Photosystem II function (dark adapted *F*_*v*_ */F*_*m*_) for: (**a**) *Ficus religiosa*; (**b**) *Mangifera indica*. Horizontal dotted lines indicate 50% of maximum values; the vertical dotted lines and arrows indicate T_50_ of PSII function - the temperature at which reduction in *F*_*v*_ */F*_*m*_ was 50% of the maximum values.

A four parameter logistic sigmoid curve was fitted to the chlorophyll *a* fluorescence (*F*_*v*_ */F*_*m*_) values across the range of temperatures examined using the R package ‘drc’^46^. The four parameter model with the lower asymptote set to zero was observed to generate appropriate curves. The temperature at which reduction in chlorophyll *a* fluorescence (*F*_*v*_ */F*_*m*_) was 50% of the upper asymptote (T_50_) was estimated from these curves (Fig. 2). We used 7 independent leaves from an individual at each of the temperatures to generate an *F*_*v*_ */F*_*m*_ response curve from which we estimated T_50_ for that individual. This was repeated for 3–6 replicates individuals for each species (Supplementary Table S4).

### Estimation of thermal safety margins

Thermal safety margins (TSM) for species were quantified as the difference between the upper thermal limits of leaf function (T_50_ of PSII function) and the maximum temperatures in this region, and represent the potential vulnerability of species to global warming^13, 14, 47^. We used 42.1 °C - the maximum daily air temperature recorded in this region in the last decade (GHCN - Global Historical Climatology Network daily, Version 3.22) to quantify TSM under current climatic conditions. To assess TSM for future climates we added 3 °C and 6 °C to current maximum temperatures (42.1 °C) to estimate future maximum temperatures. These represent the lower and upper predictions for increases in temperature in future tropical climates^7^.

These estimates of TSM are likely to be conservative as they assume that leaf temperatures are equal to air temperatures. While leaves can regulate temperatures to some degree via transpirational cooling, under conditions of limited water availability and exposure to full sunlight, leaf temperatures can be 5–15 °C higher than air temperature^48–51^. To better understand the consequences of potentially higher leaf temperatures, we estimated the percentage of species that will experience temperatures greater than their T_50_ for scenarios where leaf temperatures are 5 °C and 10 °C higher than the air temperatures, for current and future climates. Additionally, to understand how frequently such extreme temperatures may be experienced we estimated how many days in a year leaf temperatures exceed T_50_ for the 41 species for scenarios where leaf temperatures are 5 °C and 10 °C higher than the air temperatures, for current and future climates.

### Statistical analyses

To test for differences between evergreen and deciduous leaf habits we examined variation in thermotolerance (T_50_ of PSII function) using a mixed model ANOVA with leaf habit (evergreen and deciduous), and season (hot-dry and cool-wet) as fixed effects, and species nested within leaf habit. For this we used the 33 species for which we had estimates of thermotolerance for both the dry and rainy seasons (as mature or healthy leaves were not available for all species in both seasons). To further examine the relationship with deciduousness, we examined the correlation between the deciduousness index (DI) and thermotolerance. DI, a percentage, was converted to a proportion between 0–1 and logit transformed [log(y/(1 − y))] to better approximate normality before examination of Pearson’s correlation coefficients. Additionally, we examined the relationship between the untransformed DI and thermotolerance with Spearman’s rank correlations.

LMA and LA were log transformed to meet assumptions of normality. Relationships with thermotolerance were analyzed using Pearson’s correlations for the transformed, and with Spearman’s rank correlations for the untransformed variables. Estimates for thermotolerance, LMA and leaf area obtained during the dry season (35 species) were used for these analyses except for 2 species for which leaves were only available for the wet season. We did not include 4 species for which we did not have estimates of both leaf traits and thermotolerance in the same season. We also conducted these analyses separately for the dry and rainy season.

To test for seasonal changes in thermotolerance, we examined variation in the paired differences (within individuals) between dry season and rainy season thermotolerance with a mixed model ANOVA with leaf habit (evergreen and deciduous) as a fixed effect, and species nested within leaf habit. For this we used the 33 species for which we had estimates of thermotolerance for both the dry and rainy seasons. All analyses were performed using Statistica (version 9.1, Statsoft, Tulsa, OK, USA).

## Results

### The temperature response of PSII function

Dark adapted chlorophyll *a* fluorescence (*F*_*v*_ */F*_*m*_) did not change between 25 °C and 40 °C. There was a sharp decline in *F*_*v*_ */F*_*m*_ after 40 °C, and at 50 °C this was reduced to near zero for the more sensitive species (Fig. 2; for additional representative temperature response curves see Supplementary Fig. S3). For species that were more tolerant to high temperatures *F*_*v*_ */F*_*m*_ was not reduced to zero even at 52.5 °C, the highest temperatures examined. The temperature at which *F*_*v*_ */F*_*m*_ was reduced to 50% of the control values (T_50_, Fig. 2) ranged from 45.5 °C in the most sensitive, to 50.5 °C in the most thermotolerant species.

### Relationship between thermotolerance and deciduousness

**Figure 3:**
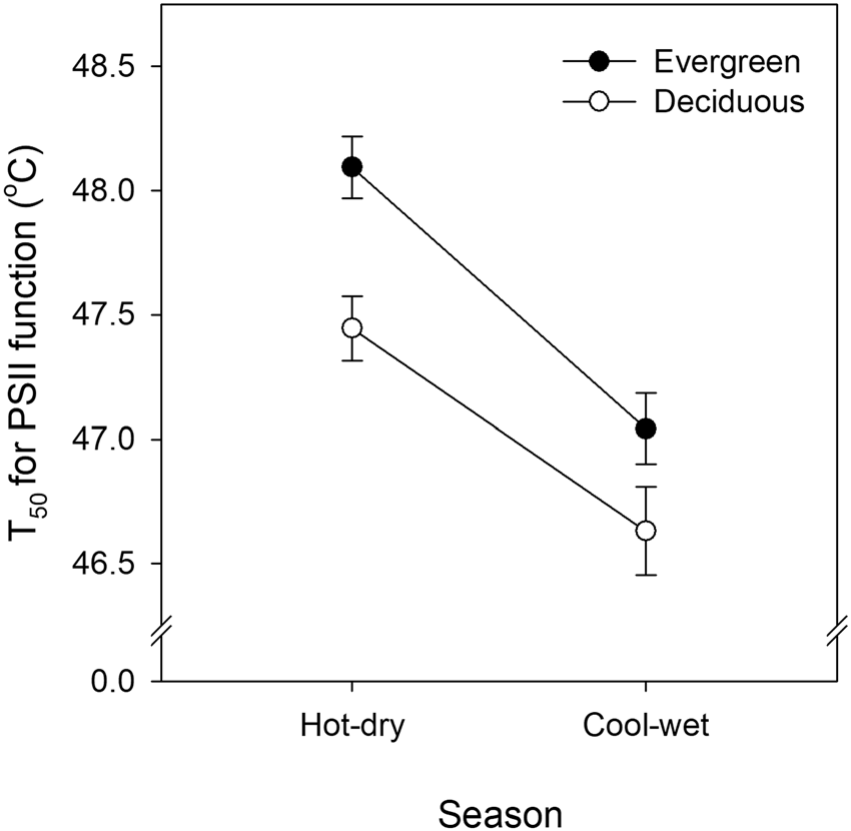
Thermotolerance in evergreen (closed circles; n = 16) and deciduous species (open circles; n = 17) in the hot-dry and the cool-wet rainy season. Error bars represent ± S.E. Thermotolerance measured as T_50_ of PSII function (*F*_*v*_ */F*_*m*_ - dark adapted chlorophyll *a* fluorescence).

**Figure 4:**
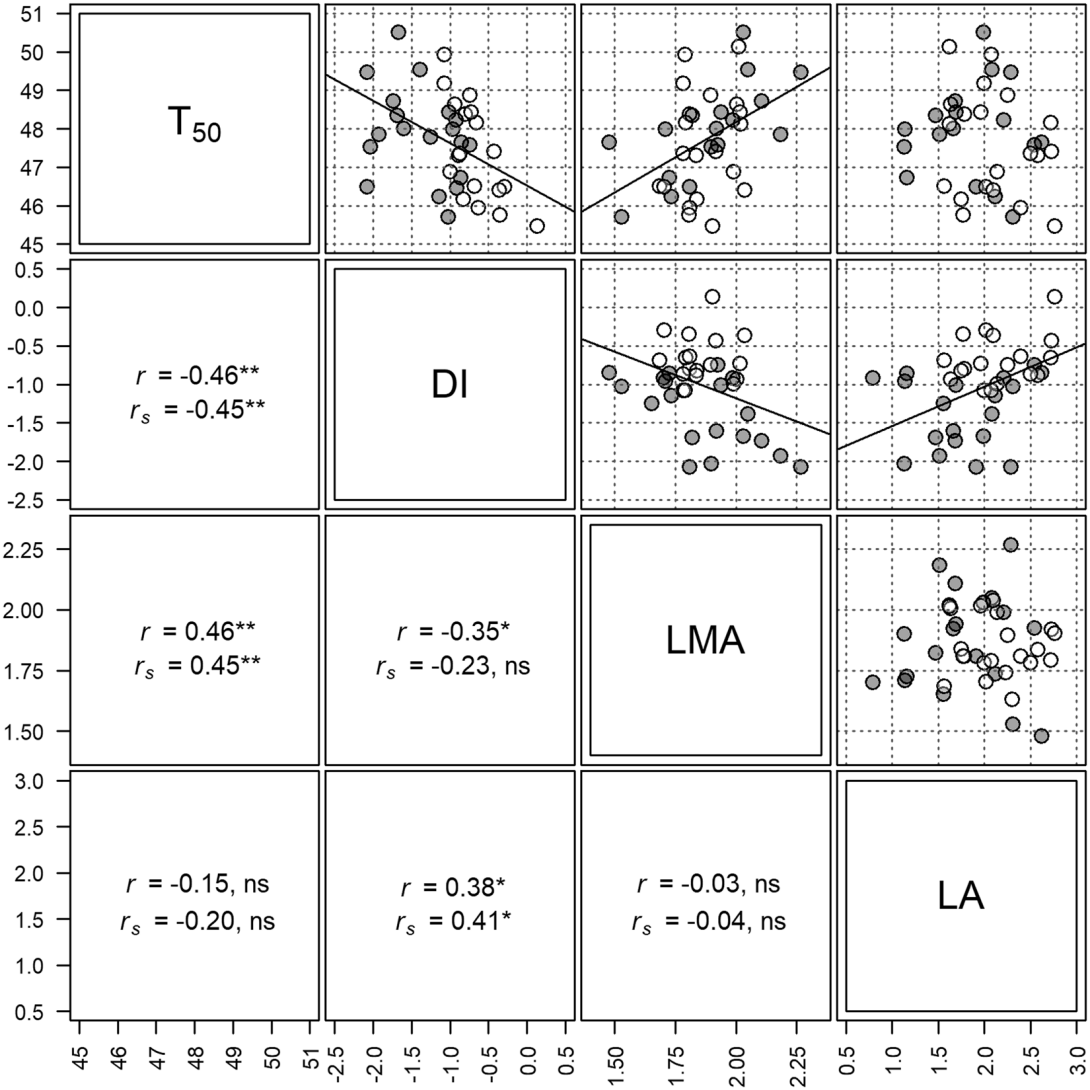
Relationship between thermotolerance, deciduousness and leaf traits. Scatter-plots, Pearson’s correlation coefficients (*r*), and Spearman’s rank correlations (*r*_*s*_) for thermotolerance (T_50_, °C), deciduousness index (DI, %), leaf mass per area (LMA, g·m^−2^), leaf size (LA, cm^2^). LMA and LA were log transformed, and DI was converted to a proportion and logit transformed to better approximate normality. Spearman’s rank correlations are for untransformed variables. Best fit lines were plotted using type II ordinary least squares linear regressions. Evergreen and deciduous species are represented by grey and open circles respectively; ns - not significant, *p < 0.05, **p < 0.01.

Evergreen species had higher thermotolerance than deciduous species in both the hot-dry pre-monsoon and the cool-wet monsoon season (Fig. 3, Table 1). The index of deciduousness (DI) provided a continuous estimate of leafing behaviour that ranged from less than 1% for evergreen species that did not display any significant loss of leaves through the year, to 58% for the most deciduous species (Table S3). Thermotolerance was negatively related to the deciduousness index (Fig. 4, Supplementary Table S5) indicating that more deciduous species with greater loss of leaves through the year exhibited lower thermotolerance. This is congruent with the previous result that showed higher thermotolerance in evergreen than deciduous species when examining differences between the qualitative leaf habit categories. Spearman’s rank correlations also showed significant negative relationships between thermotolerance and DI (Fig. 4).

**Table 1:** Variation in thermotolerance (T_50_ of PSII function). Results from a mixed model ANOVA with leaf habit (evergreen and deciduous) and season (hot-dry and the cool wet rainy season) as fixed effects, and species as a random effect nested within leaf habit.

Effect	*df*	*MS*	*F*	*p*
Leaf Habit	1	20.43	30.0	<0.001
Species [Leaf Habit]	31	16.15	23.7	<0.001
Season	1	78.46	115.1	<0.001
Leaf Habit x Season	1	1.28	1.9	0.172

### Relationship between thermotolerance and leaf traits

Species with high leaf mass per area (LMA) had higher thermotolerance (Fig. 4). However, we did not observe any significant relationship between leaf size (LA) and thermotolerance (Fig. 4). The relationships between thermotolerance and leaf traits were similar when examined separately for the wet and dry seasons, i.e. significant positive relationship for LMA and lack of a relationship for leaf size (Supplementary Table S5). As LMA and LA were not normally distributed, we examined Spearman’s correlations between thermotolerance and LMA and LA, and these analyses yielded similar results (Fig. 4).

### Seasonal change in thermotolerance between the hot-dry and cool-wet seasons

**Figure 5:**
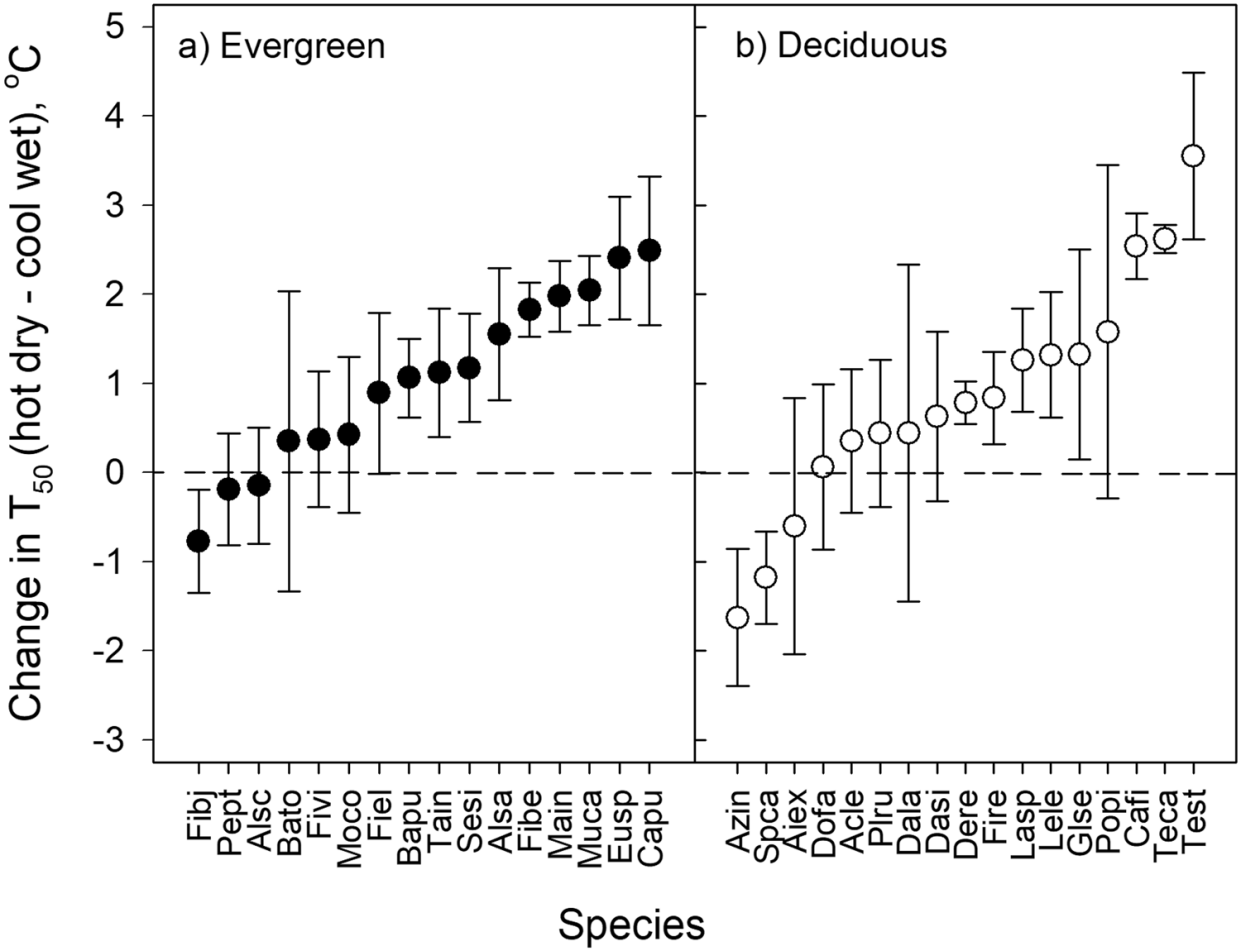
Seasonal change in thermotolerance from the hot-dry to the cool-wet season in: (**a**) evergreen, and (**b**) deciduous species. Seasonal change in T_50_ of PSII function (*F*_*v*_ */F*_*m*_ - dark adapted chlorophyll *a* fluorescence) was quantified for 3–6 individuals of each species. Species names are provided in Supplementary Table S2. Error bars represent 95% confidence intervals.

Seasonal change in thermotolerance differed among species (*F*_31,151_ = 13.779, *p* < 0.0001, Fig. 5), but not between evergreen and deciduous leaf habits (*F*_1,151_ = 2.9883, *p* = 0.0859, Fig. 5). We did not observe any significant relationships between the seasonal changes in thermotolerance and leaf traits, or the deciduous index. The change in thermotolerance between the hot dry season and the cool wet monsoon season ranged from a decrease of 3.5 °C to an increase of 1.5 °C. Of the 33 species for which we had estimates of thermotolerance in both seasons, 17 species exhibited higher thermotolerance in the hot-dry season, and we did not detect any difference for 13 species. Two deciduous and one evergreen species exhibited the opposite trend of higher thermotolerance during the monsoon season.

### Thermal safety Margins

**Figure 6:**
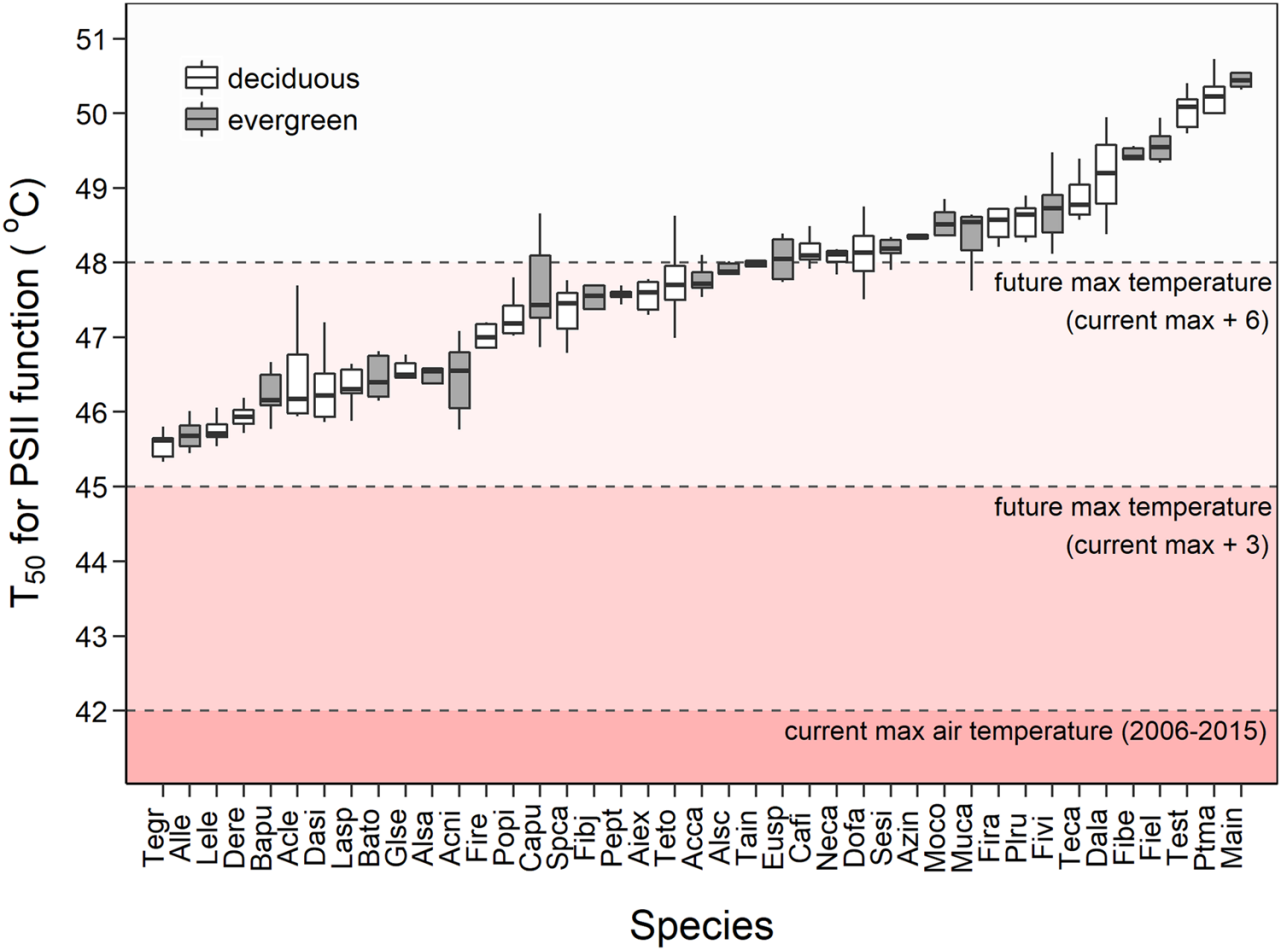
Thermotolerance (T_50_ for PSII function) for the 41 species examined. The difference between T_50_ and the three lines present a visual representation of the thermal safety margin for these species under current and future climates. The three lines represent: Current maximum daily temperatures experienced (2006–2015; data from GHCN - Global Historical Climatology Network daily, Version 3.22); Current maximum temperatures +3 °C; and Current maximum temperatures +6 °C. Boxes represent the upper 75 and lower 25 percentiles, the central line the median, and the whiskers are highest/lowest observation less than or equal to upper hinge +1.5 x inter-quartile range. Gray boxes represent evergreen and open boxes deciduous species. Details for the species are provided in Supplementary Table S2.

All species had upper thermal limits for leaf function that were greater than the highest maximum daily air temperature recorded in this region during the last ten years, and thermal safety margins (TSM) ranged from around 3.5 °C to greater than 8.5 °C (Fig. 6). In future climates with a 3 °C increase in temperature the TSM for these species is reduced, ranging from around 0.5 °C to 5.5 °C. An increase of 6 °C would result in negative TSMs for greater than 60% of the species, implying that they will experience air temperatures higher than their upper thermal limits (Fig. 6, Table 2).

**Table 2:** The percentage of species that will experience leaf temperatures greater than their T_50_, and the number of days in a year when leaf temperatures (T_leaf_) exceed T_50_. The number of days is given as a range which represents results for the most and least thermotolerant species. The distribution for all 41 species is shown in Supplementary Fig. S4. We consider three estimates of leaf temperature: i) Leaf temperatures = air temperature; ii) Leaf temperatures = air temperature + 5 °C; iii) Leaf temperatures = air temperature + 10 °C. Daily air temperature data for 10 years (2006–2015) were obtained from GHCN (Global Historical Climatology Network) daily Version 3.22. Future air temperature estimates are the upper and lower limits of the predicted increases of 3–6 °C in mean temperatures for tropical regions by the year 2100^7^.

Estimated T_leaf_	Current climate	Future climate	
		+3 °C	+6 °C
**Percentage of species examined that will experience T**_**leaf**_ ** > T**_**50**_			
i) T_leaf_ = T_air_	0	0	61
ii) T_leaf_ = T_air_ + 5 °C	32	95	100
iii) T_leaf_ = T_air_ + 10 °C	100	100	100
**Range of days in a year when T**_**leaf**_ ** > T**_**50**_			
i) T_leaf_ = T_air_	0	0	0–10
ii) T_leaf_ = T_air_ + 5 °C	0–3	0–38	10–94
iii) T_leaf_ = T_air_ + 10 °C	3–79	38–139	94–262

The above analysis assumes leaf temperatures are equal to air temperatures, but if leaf temperatures are 5 °C higher than air temperatures a third of the species examined will experience temperatures higher than T_50_ under current climatic conditions, and this will increase to 95–100% of the species in future climates with a 3–6 °C increase in temperatures (Table 2). If leaf temperatures are 10 °C higher than air temperatures all of the species examined will experience temperatures higher than their upper thermal limits under current and future climates.

Additionally, the number of days that species will be exposed to temperatures greater than T_50_ increases dramatically if leaf temperatures are higher than air temperature. With a 3 °C increase in maximum temperatures, and with leaf temperatures 5 °C higher than air temperatures, the range of days in a year that species will experience temperatures greater than T_50_ is 0 to 38 days for the most and least tolerant species, respectively (Table 2, Supplementary Fig. S4). This increases to 94 to 262 days in the most extreme scenarios where maximum temperatures increase by 6 °C and leaf temperatures are 10 °C higher than air temperatures.

## Discussion

The variation in thermotolerance and thermal safety margins observed in the 41 species examined indicate that these species may be vulnerable to, and will be differentially affected by, future warming. Importantly, variation in thermotolerance was not random, but was higher for evergreen than deciduous species, and positively related to the key functional trait, leaf mass per area. Thermotolerance was generally higher in the hot dry season, but seasonal change in thermotolerance varied among species, implying that species’ capacity to acclimate to increased temperatures may differ.

Thermotolerance (T_50_ of PSII function) in these species was similar to estimates previously reported for upper thermal limits of chlorophyll fluorescence for tropical woody species (Supplementary Table S1). However, the ~5 °C range of T_50_ among these 41 species was considerably lower than what has been reported in other tropical and temperate sites. For studies that have examined greater than 10 species, inter-specific ranges of 10–15 °C have been commonly documented^13, 17, 19^. The low thermal safety margins observed implies that these species may be particularly vulnerable to future warming, and this is congruent with what has been suggested for other tropical organisms^8^, including plants^9^.

### Higher thermotolerance in evergreen than in deciduous species

As with tolerance to other abiotic stress^25, 27^, we found that evergreen species were also more thermotolerant. Evergreen species maintain most of their canopy though the year, have longer leaf lifespan, and higher structural investment in their leaves than deciduous species^23, 26^. The longer lifespan results in exposure to a wider range of environmental conditions including extreme temperatures, and this could explain the need for higher thermotolerance. While statistically significant, the average difference in thermotolerance between evergreen and deciduous species was small, and there was substantial variation among species within leaf habit categories. The deciduousness index provided a useful continuous estimate of leafing behaviour, and confirming the lower thermotolerance observed in deciduous species compared to evergreen species, we saw a significant negative relationship between the deciduousness index and thermotolerance.

### Thermotolerance is related to the leaf economics spectrum

In support of previous studies^37, 38^, and predictions^28^, we found a positive relationship between LMA and thermotolerance. Given the central role of LMA in defining resource acquisition strategies along the “slow-fast” leaf resource acquisition spectrum^35, 36^, this suggests that fast growing productive species may be more vulnerable to future warming. Such differential vulnerabilities can result in non-random compositional shifts towards slower growing and less productive species, and could significantly alter the sink strength for atmospheric carbon and further exacerbate future climate change. Zhang *et al*. (2012) observed a positive relationship between thermotolerance and leaf longevity which is related to LMA, but did not detect a relationship with LMA. In a global analysis, O’Sullivan *et al*. (2017) did not detect a relationship between LMA and thermotolerance. Additionally, while they do not examine PSII function, a recent study did not find any relationship between the photosynthetic gas exchange responses to high temperature along the slow-fast resource acquisition spectrum in neotropical rainforest trees^52^. This raises the question whether the observed LMA-thermotolerance relationship is universal, or whether it may be specific to certain environments, e.g. hot and arid regions.

A possible explanation for the LMA-thermotolerance relationship may lie in the association between low LMA and higher photosynthetic rates with higher conductance and transpiration^36, 53^. Higher stomatal conductance and transpiration would lower leaf temperatures, and for co-occurring species this could result in lower ranges and maxima of temperatures experienced for low LMA species. Alternately, higher LMA, and associated higher structural investment and integrity may result in greater tolerance to temperatures. Due to the important implications of a LMA-thermotolerance relationship extended work on more species from diverse environments, and further research on the underlying mechanisms is warranted.

### Differential capacity for acclimation among species

We observed varied seasonal acclimation among species, including higher thermotolerance in the hot-dry season, no detectable change between the two seasons, and for 3 species, lower thermotolerance in the hot-dry season. Even within those species that showed greater thermotolerance in the hot-dry season, the magnitude of change in thermotolerance varied. This suggests that not all species will be able to acclimate equally to future warming. Some species may be particularly limited in their ability to acclimate, and therefore more vulnerable to global warming. The maximum seasonal change in T_50_ was 3.5 °C, and given that the difference in mean temperature between seasons was approximately 5 °C, the maximum acclimation rate observed was 0.7 °C per degree increase in mean habitat temperatures. These values for potential acclimation are similar to rates reported for other woody species^13^.

### Implications for global warming

The highest temperatures in this region occur at the end of the dry season when water availability is limited, and intensity of irradiance is high. The ability of leaves to effectively cool themselves via transpiration may be limited during this period, and leaf temperatures are likely to be significantly higher than air temperatures when exposed to full sunlight. Additionally, as in other dry tropical forests^54–56^, peak leaf flushing during the dry season coincides with the hottest time in the year and implies that recently flushed leaves will be exposed to extreme temperatures and are likely to suffer irreversible heat induced damage.

Future warming will represent a severe challenge for tropical forests in this region, and it is important to evaluate differential vulnerability of species to climate change. Given the complexity of tropical forests in terms of the high species diversity and environmental heterogeneity, it is not known whether these results can be generalized to other tropical forests, e.g. to the more aseasonal wet tropics, but this merits further research. Additionally, it is important to understand how interactions with other abiotic and biotic factors - elevated CO_2_^23^, nutrients, light, water availability, pest and pathogens^4^ might alter responses of tropical trees to extreme temperatures.

### Conclusion

Our results demonstrate that the upper thermal limits of these tropical trees were close to the maximum temperatures experienced, and thermal safety margins for leaf function ranged from precariously low values of 3.5 °C to around 8 °C. This implies that most of these species are likely to be severely affected by increased temperatures, and future warming will represent a major challenge for tropical trees in this region. Importantly, our results show that deciduous, and fast growing species with low leaf mass per area are more sensitive to high temperature extremes, and are therefore more likely to be more negatively affected by global warming. This differential vulnerability may lead to directional changes in species composition favouring slower growing evergreen species and such changes in species composition would alter vegetation-atmosphere feedbacks and could further exacerbate future global warming.

### Data availability

The data generated during this study are available in the published article and Supplementary Information Files.

## Electronic supplementary material

Supplementary Information
