# Supplementary material for: Leaf thermotolerance in tropical trees from a seasonally dry climate varies along the slow-fast resource acquisition spectrum
Source: Sci Rep. 2017 Sep 12;7:11246. doi: 10.1038/s41598-017-11343-5 (PMC5595873; doi:10.1038/s41598-017-11343-5)
Supplement: Supplementary file 1 — Supplementary Information [file 41598_2017_11343_MOESM1_ESM.pdf]

Supplementary Information

Leaf thermotolerance in tropical trees from a seasonally dry climate varies along the slow-fast resource acquisition spectrum

Aniruddh Sastry, Deepak Barua*

Department of Biology, Indian Institute of Science Education and Research, Pune, India

* dbarua@iiserpune.ac.in

Supplementary Table S1: Details for studies that have examined thermotolerance of tropical and sub-tropical trees, categorized by the methods used: a) LT_{50} for Leaf necrotic damage (30 min exposure); b) Critical temperature (T_c) of basal fluorescence (F_o) rise (1°C/min heating); c) T_{50} of PSII function measured by dark adapted chlorophyll fluorescence (F_v/F_m) (30 min exposure). Studies by Krause *et al.* 2010, Krause *et al.* 2013, and Krause *et al.* 2015 are included but the estimates of thermotolerance are not considered as the duration of heat treatment differ. Estimates for 2 species from O'Sullivan et al. 2017 are included in parentheses as they are exceptionally high & physiologically unrealistic.

Study	Region	No. of species	Thermotolerance (°C)
**a) Leaf tissue necrosis (LT_{50})**			
1) Lange and Lange1959	Ivory Coast	4	45 – 50
	Mauritania (desert)	16	49 – 56
	Mauritania (coast)	8	47 – 51
2) Biebl 1964	Puerto Rico	22	42 – 57
3) Karschon and Pinchas 1971	Multiple sites, Australia[1]	1	47 – 50
4) Losch 1980	Canary Islands	27	42 – 57
**b) Chlorophyll fluorescence - Critical temperature (T_c) of F_o increase**			
5) Terzaghi *et al.* 1989	Central America[2]	7	44 – 47
6) Kitao *et al.* 2000	Malaysia	4	45 – 46
7) Weng and Lai 2005	Taiwan	10	35 – 48
8) Lin 2012	Australia (multiple sites)[3]	6	47 – 49
9) Zhang *et al.* 2012	Yunnan Province, China	24	43 – 47
10) O'Sullivan et al. 2017	Northern Territory, Australia[4]	5	46 – 55
	Queensland, Australia	14	37 – 49
	Andes, Peru[5]	13	40 – 48
	Paracou, French Guiana	21	40 – 56
	Iquitos, Peru	13	38 – 57 (65, 67)
**c) Chlorophyll fluorescence - T_{50} of PSII function as measured by F_v/F_m**			
11) Larcher *et al.* 1991	Tenerife, Canary Islands	2	44 - 46
12) Yamada *et al.* 1996	Okinawa, Japan[6]	23	44
13) Cunningham and Read 2006	Australia (multiple sites)[7]	4	49 - 52
14) Krause *et al.* 2010, 2013, 2015	Panama	2	NA
15) Offord 2011	Australia (multiple sites)[8]	7	51 - 52
16) Present study	N. Western Ghats, India	41	45 - 50

[1] Study examined 3 ecotypes of *Eucalyptus camaldulensis* with tropical distributions; experiments were done in plants grown in the field in a temperate location.
[2] Study examined multiple crop and cultivated species, some of which were woody and of tropical origin; plants were grown in controlled environmental chambers.
[3] Study examined 6 *Eucalyptus* species with tropical and sub-tropical distributions. Plants were grown in a common garden at Mount Anan, Australia which has a sub-humid temperate climate.
[4] Site is geographically in the tropics, but is described as a temperate sub-humid vegetation and climate.
[5] Site is geographically in the tropics, but is a high altitude site at 3000m.
[6] Study examined tropical fruit trees grown in Okinawa Island, Japan. The data for only 1 species is shown here as for the others the methodology used was not comparable.
[7] Study examined 8 species, of which four had tropical/sub-tropical distributions. Plants were grown in a glass house where minimum temperatures were maintained above 10°C.
[8] Study examined 7 *Araucariaceae* species with tropical/sub-tropical distributions. Plants were grown in a botanical garden in Sydney, Australia which has a sub-humid temperate climate.

References (for Supplementary Table S1 and Supplementary Fig. S1)**:**

Biebl, R. 1964. Temperaturresistenz tropischer Pflanzen auf Puerto Rico. Protoplasma 59:133-156.

Cunningham, S.C. and Read, J., 2006. Foliar temperature tolerance of temperate and tropical evergreen rain forest trees of Australia. Tree Physiology 26:1435-1443.

Hijmans, R. J., L. Guarino, C. Bussink, P. Mathur, M. Cruz, I. Barrentes, E. Rojas. 2004. DIVA-GIS. V 5.0. A geographic information system for the analysis of species distribution data. Manual available at http://www.diva-gis.org.

Hijmans, R. J., S. E. Cameron, J. L. Parra, P. G. Jones, and A. Jarvis. 2005. Very high resolution interpolated climate surfaces for global land areas. International Journal of Climatology 25:1965-1978.

Karschon, R. and Pinchas, L., 1971. Variations in heat resistance of ecotypes of *Eucalyptus camaldulensis* Dehn. and their significance. Australian Journal of Botany, 19: 261-272

Kitao, M., T. T. Lei, T. Koike, H. Tobita, Y. Maruyama, Y. Matsumoto, and L.-H. Ang. 2000. Temperature response and photoinhibition investigated by chlorophyll fluorescence measurements for four distinct species of dipterocarp trees. Physiologia Plantarum 109:284-290.

Krause, G. H., K. Winter, B. Krause, P. Jahns, M. Garcia, J. Aranda, and A. Virgo. 2010. High-temperature tolerance of a tropical tree, *Ficus insipida*: methodological reassessment and climate change considerations. Functional Plant Biology 37:890-900.

Krause, G. H., A. W. Cheesman, K. Winter, B. Krause, and A. Virgo. 2013. Thermal tolerance, net CO2 exchange and growth of a tropical tree species, *Ficus insipida*, cultivated at elevated daytime and nighttime temperatures. Journal of Plant Physiology 170:822-827.

Krause, G.H., Winter, K., Krause, B. and Virgo, A., 2015. Light-stimulated heat tolerance in leaves of two neotropical tree species, *Ficus insipida* and *Calophyllum longifolium*. Functional Plant Biology, 42: 42-51.

Lange, O.L., Lange, R. (1959) Untersuchungen iiber Warmehaushalt und Hitzeresistenz mauretanischer Wasten- und Savannenpflanzen. Flora 147:595-651

Larcher, W., Wagner, J., Neuner, G., Méndez, M., Jiménez, M.S. and Morales, D., 1991. Thermal limits of photosynthetic function and viability of leaves of *Persea indica* and *Persea americana*. Acta Oecologica:(1990), 12: 529-541.

Lin, Y.S., 2012. How will *Eucalyptus* tree species respond to global climate change?--A comparison of temperature responses of photosynthesis (Doctoral dissertation, University of Western Sydney).

Lösch, R., 1980. Die Hitzeresistenz der Pflanzen des kanarischen Lorbeerwaldes. Flora, 170: 456-465.

Offord, C. A. 2011. Pushed to the limit: consequences of climate change for the *Araucariaceae*: a relictual rain forest family. Annals of Botany 108:347-357.

O'Sullivan, O.S., Heskel, M.A., Reich, P.B., Tjoelker, M.G., Weerasinghe, L.K., Penillard, A., Zhu, L., Egerton, J.J., Bloomfield, K.J., Creek, D. and Bahar, N.H., 2017. Thermal limits of leaf metabolism across biomes. Global Change Biology 23: 209-223.

Terzaghi, W. B., D. C. Fork, J. A. Berry, and C. B. Field. 1989. Low and high-temperature limits to PSII - a survey using trans-parinaric acid, delayed light-emission, and f_0 chlorophyll fluorescence. Plant Physiology 91:1494-1500.

Weng, J. H., and M. F. Lai. 2005. Estimating heat tolerance among plant species by two chlorophyll fluorescence parameters. Photosynthetica 43:439-444.

Yamada, M., T. Hidaka, and H. Fukamachi. 1996. Heat tolerance in leaves of tropical fruit crops as measured by chlorophyll fluorescence. Scientia Horticulturae 67:39-48.

Zhang, J. L., L. Poorter, G. Y. Hao, and K. F. Cao. 2012. Photosynthetic thermotolerance of woody savanna species in China is correlated with leaf life span. Annals of Botany 110:1027-1033.

Supplementary Table S2: List of species examined in the study with species code, family and whether or not the species is native to the region of study. If not native, the region of origin of the species has been mentioned.

#	Code	Species name	Family	Origin
1.	Acca	*Acacia catechu* (L.f.) Willd.	Leguminosae	Native
2.	Acle	*Acacia leucophloea* (Roxb.) Willd.	Leguminosae	Native
3.	Acni	*Acacia nilotica* (L.) Delile	Leguminosae	Tropical Africa
4.	Aiex	*Ailanthus excelsa* Roxb.	Simaroubaceae	Native
5.	Alle	*Albizia lebbeck (L.)* Benth.	Leguminosae	Native
6.	Alsa	*Albizia saman (Jacq.)* Merr.	Leguminosae	Malaysia
7.	Alsc	*Alstonia scholaris* (L.) R. Br.	Apocynaceae	Native
8.	Azin	*Azadirachta indica* A. Juss.	Meliaceae	Native
9.	Bapu	*Bauhinia purpurea* L.	Leguminosae	Native
10.	Bato	*Bauhinia tomentosa* L.	Leguminosae	Native
12.	Capu	*Caesalpinia pulcherrima* (L.) Sw.	Leguminosae	Native
13.	Cafi	*Cassia fistula* L.	Leguminosae	Native
14.	Dala	*Dalbergia lanceolaria* L.f.	Leguminosae	Native
15.	Dasi	*Dalbergia sissoo* DC.	Leguminosae	Native
16.	Dere	*Delonix regia* (Hook.) Raf.	Leguminosae	Madagascar
17.	Dofa	*Dolichandrone falcata* (Wall. ex DC.) Seem.	Bignoniaceae	Native
18.	Eusp	*Eucalyptus* sp.	Myrtaceae	Australia
19.	Fibe	*Ficus benghalensis* L.	Moraceae	Native
20.	Fibj	*Ficus benjamina* L.	Moraceae	Native
21.	Fiel	*Ficus elastica* Roxb. ex Hornem.	Moraceae	Native
22.	Fira	*Ficus racemosa* L.	Moraceae	Native
23.	Fire	*Ficus religiosa* L.	Moraceae	Native
24.	Fivi	*Ficus virens* Aiton	Moraceae	Native
25.	Glse	*Gliricidia sepium* (Jacq.) Walp.	Leguminosae	Central America
26.	Lasp	*Lagerstroemia speciosa* (L.) Pers.	Lythraceae	Native
27.	Lele	*Leucaena leucocephala* (Lam.) de Wit	Leguminosae	Mexico
27.	Main	*Mangifera indica* L.	Anacardiaceae	Native
28.	Moco	*Morinda coreia* Buch.-Ham.	Rubiaceae	Native
29.	Muca	*Muntingia calabura* L.	Muntingiaceae	Jamaica
30.	Neca	*Neolamarckia cadamba* (Roxb.) Bosser	Rubiaceae	Native
31.	Pept	*Peltophorum pterocarpum* (DC.) K.Heyne	Leguminosae	South-east Asia
32.	Plru	*Plumeria rubra* L.	Apocynaceae	Central America
33.	Popi	*Pongamia pinnata* (L.) Pierre	Leguminosae	Native
34.	Ptma	*Pterocarpus marsupium* Roxb.	Leguminosae	Native
35.	Sesi	*Senna siamea* (Lam.) H.S.Irwin & Barneby	Leguminosae	Native
36.	Spca	*Spathodea campanulata* P.Beauv.	Bignoniaceae	Africa
37.	Tain	*Tamarindus indica* L.	Leguminosae	Tropical Africa
38.	Test	*Tecoma stans* (L.) Juss. ex Kunth	Bignoniaceae	Central America
39.	Tegr	*Tectona grandis* L.f.	Lamiaceae	Native
40.	Teto	*Terminalia tomentosa* Wight & Arn	Combretaceae	Native
41.	Teca	*Terminalia catappa* L.	Combretaceae	Native

Supplementary Table S3: Species code (Sp - details in Supplementary Table S2), leaf habit (LH: E- evergreen, D - deciduous), height (range in m), deciduousness index (DI, %), month of peak leaf flushing (FL), month of peak leaf senescence (SEN). NA indicates phenology not monitored. SE - standard error, n - sample size.

Sp	Leaf Habit	n	Height	DI (%)	SE	FL	SEN
Acca	E	8	3-5	5.31	0.47	Apr	Dec
Acle	D	10	5-8	17.08	0.26	May	Jan
Acni	E	5	8-12	10.83	2.04	Apr	Mar
Aiex	D	10	10-15	27.08	0.62	Apr	Jan
Alle	E	10	10-20	8.58	0.55	Mar	Dec
Alsa	E	10	10-15	0.83	0	May	Dec
Alsc	E	10	8-12	2.42	0.19	Apr	Feb
Azin	D	10	10-15	13.67	1.07	Apr	Jan
Bapu	E	10	6-12	6.67	0.37	Apr	Jan
Bato	E	10	3-5	12.17	0.62	Jun	Jan
Cafi	D	10	8-12	18.08	1.92	Apr	Dec
Capu	E	10	5-8	12.33	0.3	Apr	Dec
Dala	D	4	10-15	7.71	0.52	Jun	May
Dasi	D	10	8-12	13.04	0.82	May	Jan
Dere	D	10	10-15	18.83	0.43	Mar	Jan
Dofa	D	NA	5-8	--	--	--	--
Eusp	E	10	10-15	1.17	0.31	Apr	Jan
Fibe	E	10	10-15	3.92	0.35	Jun	Apr
Fibj	E	10	10-15	0.92	0.34	Jan	May
Fiel	E	4	10-15	0.83	0.41	Jan	Jan
Fira	D	10	10-15	10.33	0.42	Oct	Sep
Fire	D	10	10-15	9.17	0.33	Jun	Apr
Fivi	E	6	10-15	1.8	0.14	Apr	Dec
Glse	D	10	10-15	33.67	0.55	May	Jan
Lasp	D	10	8-12	30.25	0.48	May	Apr
Lele	D	10	10-15	31	1.05	May	Jan
Main	E	10	10-15	2.08	0.14	May	Dec
Moco	E	10	8-10	8.83	0.36	Apr	Dec
Muca	E	10	5-8	2	0.31	Apr	Apr
Neca	D	NA	10-15	--	--	--	--
Pept	E	10	10-15	15.25	0.51	Apr	Dec
Plru	D	10	3-5	15.75	0.61	Apr	Dec
Popi	D	4	8-10	11.46	0.52	Apr	Feb
Ptma	D	NA	10-15	--	--	--	--
Sesi	E	10	10-15	10.75	0.49	Apr	Dec
Spca	D	6	10-15	11.94	2.45	Apr	Jan
Tain	E	4	10-15	9.79	0.4	May	Jan
Teca	D	10	8-10	15.29	0.8	Jan	Apr
Tegr	D	10	10-15	57.75	0.43	Mar	Dec
Test	D	10	5-8	7.75	0.4	Jun	Apr
Teto	D	NA	10-15	--	--	--	--

Supplementary Table S4: Species code (Sp - details in Supplementary Table 2), leaf mass per area - dry season (disc LMA-d, g·m^{-2}), leaf size - dry season (LA-d, cm^{2}), leaf mass per area - rainy season (disc LMA-r, g·m^{-2}), leaf size - dry season (LA-r, cm^{2}), T_{50} - hot dry season (T_{50}-d, °C), T_{50} - rainy season (T_{50}-r, °C). NA indicates that healthy mature leaves for that species not available or otherwise not measured. SE - standard error, n - sample size.

Sp	LMA-d	SE	LA-d	SE	n	LMA-r	SE	LA-r	SE	n	T50-d	SE	n	T50-r	SE	n
Acca	NA	--	36	2.2	--	45	1.4	NA	--	5	47.8	0.1	6	NA	--	--
Acle	48.3	0.9	36.9	2.4	5	47.4	1.3	42	8.6	5	46.5	0.3	5	46.2	0.2	6
Acni	NA	--	6.2	0.4	--	50.2	3.2	NA	--	5	46.4	0.2	5	NA	--	--
Aiex	82.8	1	535.3	43.3	5	62.4	3.8	996.9	152.7	5	47.4	0.5	6	48	0.8	6
Alle	NA	--	NA	--	--	33.8	1.8	205.1	51.3	2	NA	--	--	45.7	0.1	6
Alsa	64.4	1.4	82.2	2.7	5	76.3	1.8	59.7	8	5	46.5	0.3	5	45	0.2	6
Alsc	44.2	0.6	45.9	1.8	5	83.3	6.9	56.9	6.8	5	47.9	0.1	6	48	0.2	6
Azin	64.4	5.6	61.4	2.1	5	91.2	3.1	140.4	35.5	4	48.4	0.1	6	50	0.3	6
Bapu	54.3	2.7	130.8	11.3	5	45.9	4.4	109.7	8.4	5	46.2	0.1	6	45.2	0.1	6
Bato	53.2	1.3	14.6	0.6	5	52.9	6.3	15.7	1.6	5	46.7	0.4	6	46.4	0.4	6
Cafi	62.1	1.5	524.8	20.8	5	NA	--	530.8	55.5	--	48.2	0.1	6	45.7	0.1	4
Capu	30.1	2.3	416.8	18.3	5	33.1	0.3	408	24.6	5	47.7	0.3	6	45.2	0.4	6
Dala	60.5	2.9	100.1	5.8	5	62.2	3.3	42.3	3.2	5	49.2	0.5	3	48.7	0.5	3
Dasi	68.9	1.2	55.9	2.3	5	80.9	2.6	61.8	2.9	5	46.2	0.3	6	45.5	0.4	6
Dere	64.5	2	247.7	9.9	5	66.8	0.9	248	13.6	5	45.9	0.1	6	45.2	0.1	6
Dofa	104.5	2.9	41.8	1.7	5	NA	--	NA	--	--	48.1	0.2	6	48.1	0.3	6
Eusp	152.5	4.6	32.7	1.5	5	164.2	7.4	37.1	2.3	5	47.8	0.3	6	45.4	0.1	6
Fibe	111.8	3.1	122	6.3	5	118.7	6.9	73.5	5.7	5	49.5	0.2	6	47.7	0.1	6
Fibj	79.3	1.3	13.5	0.3	5	95.9	5.1	14.7	0.7	5	47.5	0.2	6	48.3	0.2	6
Fiel	185	4.6	193.3	8.5	5	171.6	7.9	151.7	22.5	5	49.5	0.2	6	48.6	0.3	6
Fira	101.1	6.5	43.3	1.7	5	NA	--	NA	--	--	48.6	0.2	6	NA	--	--
Fire	97.6	3.1	138.8	4	5	83.1	1.2	89.7	10.7	5	46.9	0.2	6	46	0.1	6
Fivi	127.9	2.3	48.5	1.2	5	136.2	6.9	43.8	3	5	48.7	0.2	6	48.3	0.1	6
Glse	50.6	1.2	104.5	3.9	5	40.8	1.6	151	7.8	5	46.5	0.1	6	45.2	0.4	6
Lasp	108.9	6	124.6	9.7	5	88	1.7	149.7	9.4	5	46.4	0.2	6	45.1	0.1	6
Lele	64.2	5.4	58.7	3	5	64.8	2.1	60	7.1	5	45.8	0.1	6	44.4	0.2	6
Main	107.1	1.8	97.6	4.1	5	113.6	5	82	2.6	5	50.5	0.1	6	48.5	0.1	6
Moco	87	1.4	49.6	1.9	5	102.5	4.3	49.9	13.1	5	48.4	0.2	6	48	0.2	6
Muca	66.3	2.8	29.9	1.2	5	56.5	3.1	35	2.3	5	48.3	0.2	6	46.3	0.1	6
Neca	NA	--	NA	--	--	42.6	1.9	201.7	32.6	5	48.1	0.1	4	NA	--	--
Pept	84	2.7	349.8	34.2	5	80.2	2.4	292	36	5	47.6	0	5	47.8	0.2	6
Plru	104	7.6	91.9	3.3	5	110.3	6	112.1	4.4	5	48.4	0.2	6	48	0.3	6
Popi	68.4	1.6	380.9	45.4	5	69.7	3.9	185	14.8	5	47.3	0.2	4	45.7	0.6	4
Ptma	103	2.1	42.1	1.6	5	NA	--	NA	--	--	50.1	0.3	4	NA	--	--
Sesi	97.4	2.5	163.2	5.9	5	92.5	7.6	202.7	9.7	5	48.2	0.1	6	47.1	0.1	5
Spca	60.5	6.3	NA	--	5	NA	--	319.9	36.3	--	47.3	0.2	6	48.5	0.1	6
Tain	51.3	1.6	13.9	0.8	5	53.4	3.3	12	2	5	48.0	0	4	46.9	0.3	4
Teca	78.5	2.5	178.2	8.9	5	NA	--	NA	--	--	48.9	0.1	6	46.3	0.2	5
Tegr	NA	--	NA	--	--	80	1.6	580.6	46.1	5	NA	--	--	45.5	0.2	6
Test	61.7	3	118.9	4.3	5	55.1	3.1	201.2	21.4	5	49.9	0.2	6	46.4	0.2	6
Teto	NA	--	NA	--	--	55	--	171	--	1	47.8	0.3	4	NA	--	--

Supplementary Table S5: Relationship between thermotolerance (T_{50} for PSII function) and: a) Deciduousness index (DI, %); b) Leaf mass per area (LMA, $g \cdot m^{-2}$), and c) Leaf area (LA, cm^2). These analyses were conducted for the hot-dry and the cool-wet rainy season separately. Values for Pearson's correlation coefficient (Logit transformed DI, log transformed LMA and LA to meet assumptions of normality), and Spearman's rank correlation (untransformed) are shown.

		**Pearson's**		**Spearman's**	
	**n**	***r***	***p***	r_s	***p***
**a) Deciduousness Index (DI)**					
dry season	35	-0.41	0.014	-0.44	0.008
rainy season	34	-0.32	0.066	-0.35	0.040
**b) Leaf mass per area (LMA)**					
dry season	35	0.41	0.013	0.41	0.015
rainy season	31	0.48	0.006	0.46	0.010
**c) Leaf area (LA)**					
dry season	36	0.07	0.685	0.01	0.994
rainy season	33	-0.11	0.560	-0.13	0.455

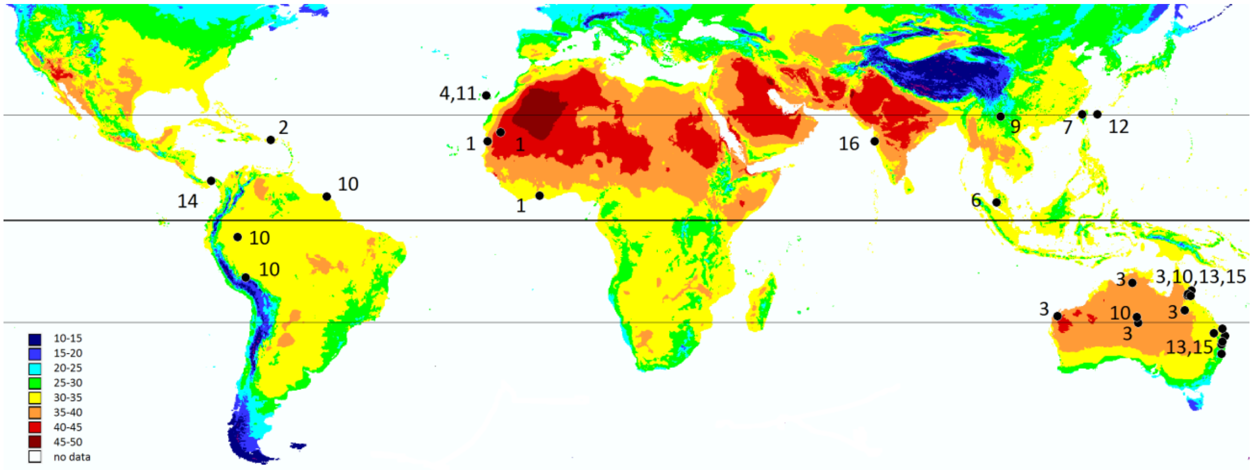

Supplementary Figure S1: Geographic coverage of studies that have examined temperature tolerance in tropical and sub-tropical trees. Circles represent locations of studies (details presented in Supplementary Table S1). For studies 7 and 12, crops and cultivated species of tropical origin were examined. For studies 3, 13 and 15 sites of origin for the species or ecotypes examined are shown. For these, experimental plants were grown in a common environment at a separate site (not shown). The bold line represents the equator, and the thinner lines the tropics of Cancer and Capricorn. The above map was generated using DIVA-GIS V.5.0 (Hijmans *et al*. 2004). The background colours represent the hottest temperatures of the year (in °C; data from Worldclim, Hijmans *et al*. 2005).

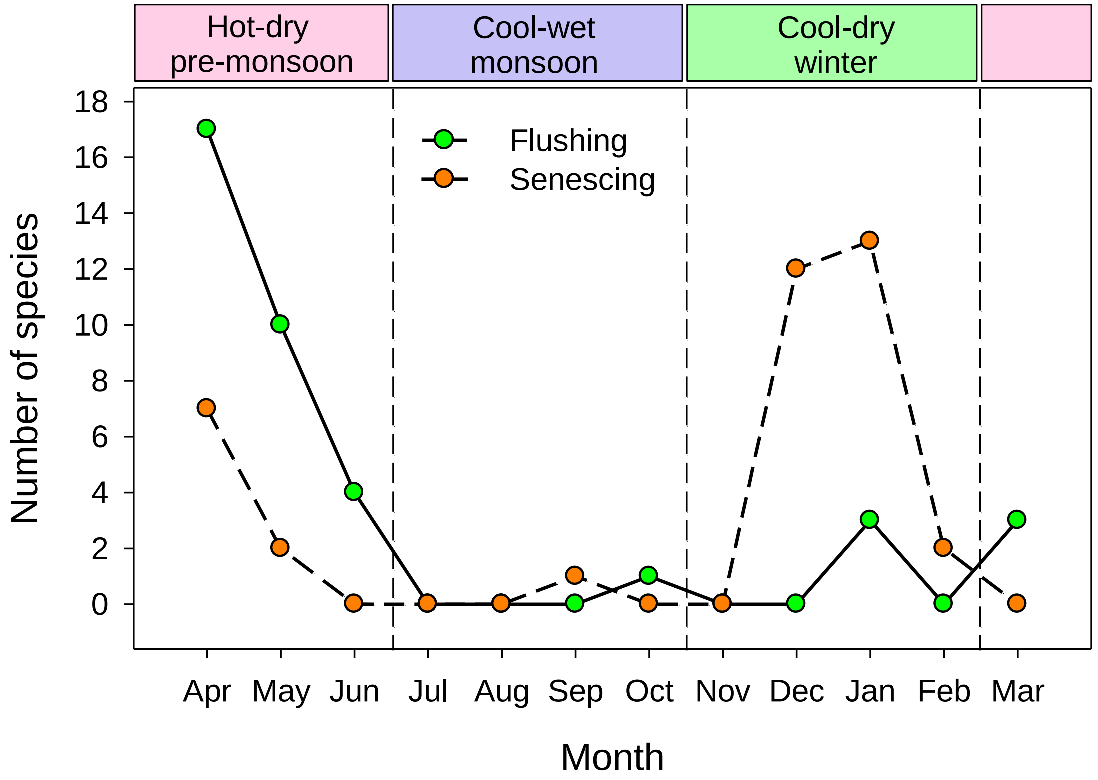

Supplementary Figure S2: Leaf flushing and senescing patterns. The number of species in peak leaf flushing (green) and senescing (orange) for the 37 species for which leaf phenology was monitored between April 2014 and March 2015.

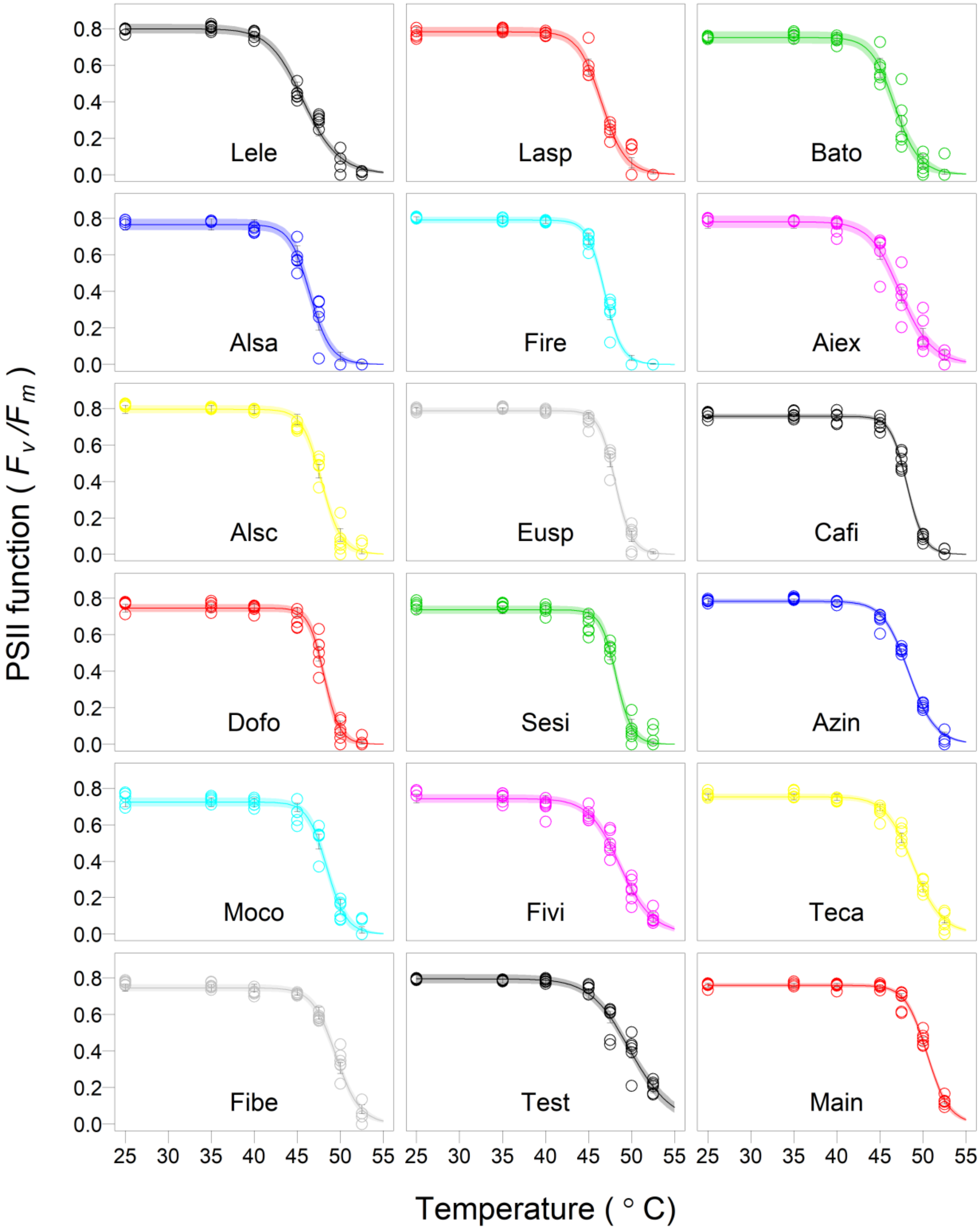

Supplementary Figure S3: Representative species level temperature response curves for F_v/F_m during the dry season spanning the range of thermotolerance observed (arranged from low to high thermotolerance as determined by the temperature at which reduction in F_v/F_m was 50% of the maximum - T_{50} of PSII function). Details for species provided in Supplementary Table S2.

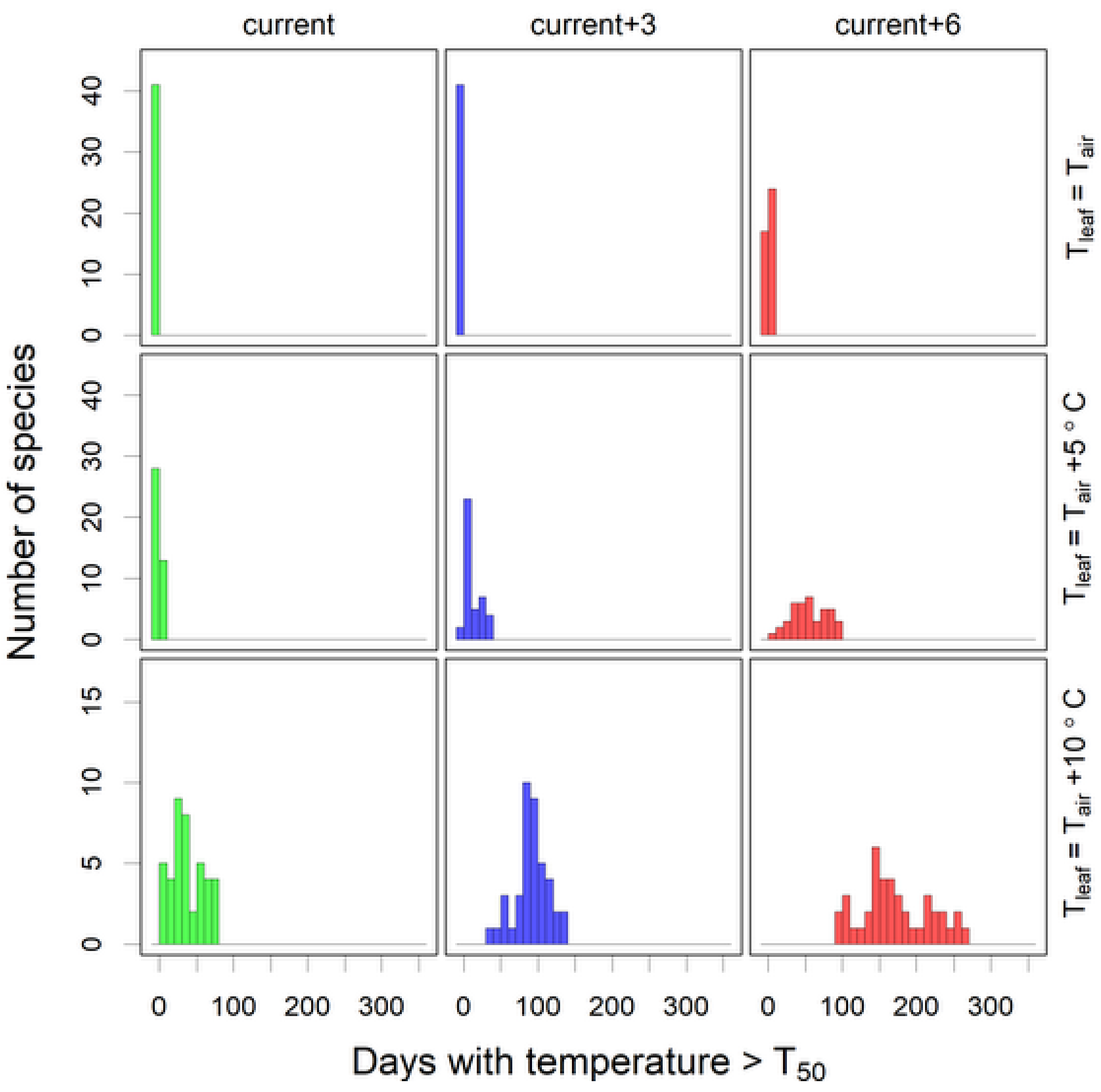

Supplementary Figure S4: Distribution of the number of days where leaf temperature (T_{leaf}) exceeds thermotolerance (T_{50} of PSII function) for the 41 study species in current and future climates. We consider three estimates of leaf temperature for a given air temperature (T_{air}): $T_{leaf} = T_{air}$; $T_{leaf} = T_{air}$ + 5°C; $T_{leaf} = T_{air}$ + 10°C. Daily air temperature data for 10 years (2006-2015) were obtained from GHCN (Global Historical Climatology Network) daily Version 3.22. Future air temperature estimates are the upper and lower limits of the predicted increases of 3-6°C in mean temperatures for tropical regions by the year 2100 (Malhi *et al.* 2014).
